# On the activity loss of hydrolases in organic solvents: II. a mechanistic study of subtilisin Carlsberg

**DOI:** 10.1186/1472-6750-6-51

**Published:** 2006-12-22

**Authors:** Betzaida Castillo, Vibha Bansal, Ashok Ganesan, Peter Halling, Francesco Secundo, Amaris Ferrer, Kai Griebenow, Gabriel Barletta

**Affiliations:** 1 Department of Chemistry, University of Puerto Rico, Rio Piedras Campus, P.O Box 23346, San Juan, 00931-3346, Puerto Rico; 2 Department of Chemistry, University of Puerto Rico at Humacao, CUH Station, Humacao, 00791, Puerto Rico; 3 WestCHEM, Department of Pure & Applied Chemistry, University of Strathclyde, Glasgow G1 1XL, UK; 4 Istituto di Chimica del Riconoscimento Molecolare, CNR, Via Mario Bianco 9, 20131 Milano, Italy

## Abstract

**Background:**

Enzymes have been extensively used in organic solvents to catalyze a variety of transformations of biological and industrial significance. It has been generally accepted that in dry aprotic organic solvents, enzymes are kinetically trapped in their conformation due to the high-energy barrier needed for them to unfold, suggesting that in such media they should remain catalytically active for long periods. However, recent studies on a variety of enzymes demonstrate that their initial high activity is severely reduced after exposure to organic solvents for several hours. It was speculated that this could be due to structural perturbations, changes of the enzyme's pH memory, enzyme aggregation, or dehydration due to water removal by the solvents. Herein, we systematically study the possible causes for this undesirable activity loss in 1,4-dioxane.

**Results:**

As model enzyme, we employed the protease subtilisin Carlsberg, prepared by lyophilization and colyophilization with the additive methyl-β-cyclodextrin (MβCD). Our results exclude a mechanism involving a change in ionization state of the enzyme, since the enzyme activity shows a similar pH dependence before and after incubation for 5 days in 1,4-dioxane. No apparent secondary or tertiary structural perturbations resulting from prolonged exposure in this solvent were detected. Furthermore, active site titration revealed that the number of active sites remained constant during incubation. Additionally, the hydration level of the enzyme does not seem to affect its stability. Electron paramagnetic resonance spectroscopy studies revealed no substantial increase in the rotational freedom of a paramagnetic nitroxide inhibitor bound to the active site (a spin-label) during incubation in neat 1,4-dioxane, when the water activity was kept constant using BaBr_2 _hydrated salts. Incubation was also accompanied by a substantial decrease in V_max_/K_M_.

**Conclusion:**

These results exclude some of the most obvious causes for the observed low enzyme storage stability in 1,4-dioxane, mainly structural, dynamics and ionization state changes. The most likely explanation is possible rearrangement of water molecules within the enzyme that could affect its dielectric environment. However, other mechanisms, such as small distortions around the active site or rearrangement of counter ions, cannot be excluded at this time.

## Background

Enzymes have been successfully employed to catalyze a number of transformations and chiral resolutions of biological and industrial importance in organic solvents [1-10]] It is well documented that enzyme catalysis in these non-natural media offers some advantages over that in natural-aqueous environment, such as: prevention of autolysis (in case of proteases) and increased thermostability [11,12]. In fact, it has been shown that enzymes can perform catalysis in organic solvents at temperatures far above those temperatures that denature enzymes in aqueous systems [8]. Despite this, enzyme inactivation in organic solvents has been reported, and studies have addressed this issue. For example, Fagain et al. (2003) have recently reviewed bioreactor stability, shelf life and operational stability of a variety of enzymes suspended in neat organic solvents and aqueous-organic solvent mixtures [13].

We have previously reported that the activity of the serine protease subtilisin Carlsberg is significantly reduced upon storage in several organic solvents, irrespective of its preparation, hydration, hydrophobicity of the solvent, the reaction temperature and of the substrates used [14]. A subsequent study using various lipases, an esterase, and α-chymotrypsin showed that for these enzymes most of their initial high activity also plunges upon exposure to organic solvents [15]. It was found that some additives and modes of enzyme preparation (such as chemical modification with polyethylene glycol) were able to reduce activity loss of the enzymes in various organic solvents. The other variables studied (enzyme hydration, the reaction temperature, the organic solvent and the enzymes themselves) were not found to affect the rapid loss of activity observed. Furthermore, experiments using structurally defined cross-linked enzyme crystals (CLECs) [11,16] suggested that the mechanism of inactivation might not involve large structural changes of the enzyme catalyst. The activity loss was reversible upon re-lyophilizing the enzyme from an aqueous buffer, indicating that inactivation did not involve autolysis or irreversible denaturation of the enzyme [14].

Those findings outline a clear drawback in the use of biocatalysts for applications that require prolonged exposure to organic solvents, and showed the need for understanding the mechanism of enzyme inactivation during storage in these media. Herein we study this detrimental effect in detail using two different preparations of the model enzyme subtilisin Carlsberg.

## Results

The results previously reported from this and other laboratories demonstrated that most enzymes lose their initial high activity exponentially during the first hours of incubation in a variety of organic solvents until a constant activity value is reached. So far, all of the enzymes studied show some residual activity after a long incubation period (usually from 4 to 7 days), which appears to persist indefinitely [14,15,17]. However, no mechanistic explanation for this phenomenon has been provided so far. Herein we examine the structure, the flexibility, the number of active sites remaining, the powder morphology, and the pH profile of subtilisin Carlsberg as some of the possible causes that could contribute to the observed decrease in enzyme activity after prolonged exposure to organic media. We had previously demonstrated that subtilisin Carlsberg instability was independent of: (a) the mode of enzyme preparation (lyophilized, co-lyophilized and CLECs – all showed a similar stability profile); (b) the physicochemical properties of the solvents used; and (c) the substrates [14]. Subsequent studies involving different preparations of α-chymotrypsin, pig-liver esterase and several lipases showed that this was not unique to subtilisin, but rather a common effect shared by several enzymes [15].

First, we decided to study the enzyme-powder morphology after suspension in 1,4-dioxane since it has been suggested that incubation could result in larger and more compact aggregates, which could reduce the enzyme activity.

### Enzyme morphology

**Figure 1:**
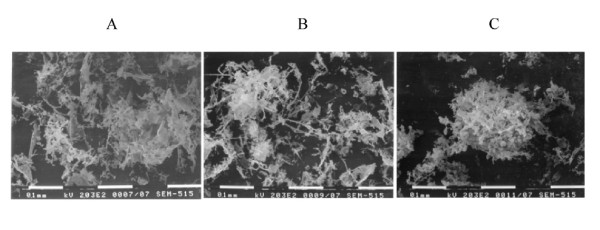
Scanning electron microscopy (SEM) of co-lyophilized subtilisin C. in 1,4-dioxane. Incubation times in 1,4-dioxane: (A) 10 minutes; (B) 3 hrs; (C) 4 days. The color segments of the scale bars (shown on the figures) are of 0.1 mm.

Since most of the enzyme preparations studied were suspensions, and therefore the substrates would have to diffuse through solution and through the pores of solid particles to reach their corresponding active sites, it is conceivable that particle aggregation or morphology changes (such as shrinking or powder deformation) could have an adverse effect on the substrate's diffusion to the enzyme's active site. Scanning Electron Microscopy (SEM) of the suspended particles of the co-lyophilized enzyme in 1,4-dioxane showed that no apparent morphological changes or aggregation occurred during incubation (Figure 1). The typical flaky suspended particles remained nearly of the same size, shape, and morphology, suggesting that this is probably not the cause for the activity drop. In agreement with these results, a soluble preparation of the same enzyme, for which its activity should not be compromised by aggregation or morphological changes, also showed a decrease in activity during incubation, similar to what was observed with their suspended counterparts [15]. From these results we can rule out substrate diffusion limitations as a possible cause for the observed decrease in enzyme activity.

### Active-site titration and Michaelis constant

Next, as a likely cause for this effect, we decided to determine if the fraction of active enzyme molecules decreases during incubation. Active-site titration, employing the method described by Wangikar et al., [18] of the lyophilized powder before and after incubation in 1,4-dioxane, at 45°C, showed no change in the number of competent active-sites: 17 ± 2% before incubation and 20 ± 2% after incubation. The apparent V_max _calculated for the co-lyophilized preparation in 1,4-dioxane was substantially reduced after a 4-day incubation period. The apparent Michaelis constant (K_M_) calculated for the co-lyophilized preparation increased substantially after the incubation period (Table 1). Furthermore, the V_max_/K_M _showed a substantial decrease after incubation (Table 1). For Ping-Pong kinetics, a true value of V_max_/K_M _is obtained even when only one substrate concentration is varied (as in our experiments), so it seems that the observed drop in activity is due to a deficiency in the catalytic efficiency of the enzyme. The increase in the K_M _value could indicate subtle structural changes of surface amino groups involved in the substrate binding process. Such subtle (reversible) structural changes of active site residues could also explain decreased catalytic efficiency of the catalyst.

### Structural analysis

**Figure 2:**
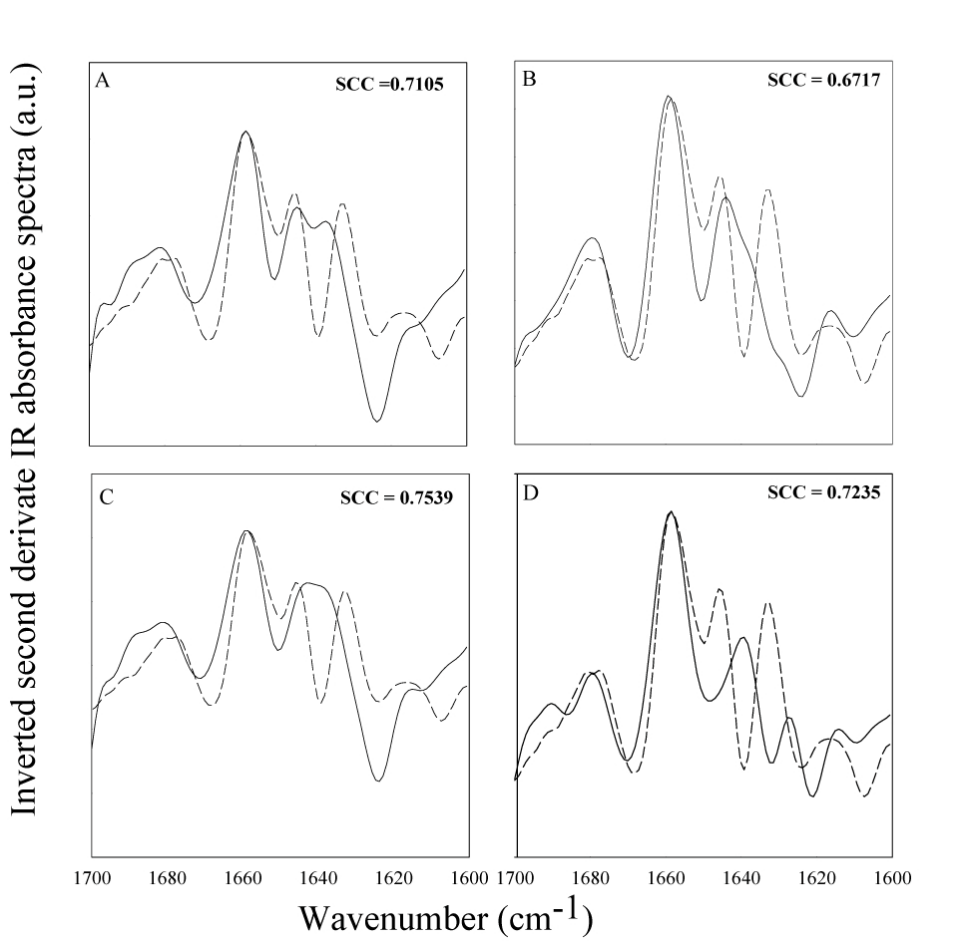
Inverted second derivative absorbance spectra of subtilisin: in aqueous medium (dashed line) and after suspension in 1,4-dioxane (solid line) at different times of incubation. Lyophilized powder before incubation (A), and after incubation for 4 days (B). Co-lyophilized enzyme before (C), and after incubation for 4 days (D).

It is well documented that the dehydration step, required before introducing an enzyme to the organic medium (and usually achieved by lyophilization), induces structural perturbation in the enzyme's secondary structure [12,19]. It has also been shown that subsequent introduction to organic media (aprotic organic solvents) does not induce additional structural perturbations in an enzyme (since an enzyme suspended in such medium would not be able to rearrange its hydrogen bonds, and it will therefore be structurally stable) [20,21]. However, it is possible that structural perturbations or partial denaturation resulting from water removal, solvent insertion, or protein relaxation could take place over a prolonged exposure to neat organic solvents, and this might explain our observed-reduced enzyme activity after incubation. Secondary structure analysis of the lyophilized and the co-lyophilized powders before and after incubation in 1,4-dioxane by Fourier Transform Infrared Spectroscopy (FTIR) revealed no significant secondary structural changes. The SCC of the lyophilized powder before incubation was found to be 0.7105 (determined with respect to the native enzyme) and 0.6717 after incubation (yielding a negligible difference of 0.0388). Similarly, the SCC of the co-lyophilized enzyme before and after incubation were nearly identical (0.7539 and 0.7235, (Figure 2). Furthermore, the inverted second derivative absorbance spectra in dry state and in a different solvent, THF, are also nearly identical. These results suggest that no secondary changes or extensive autolysis occurs during incubation in these solvent, in agreement with our active-site titration results and previous findings [14,15].

**Figure 3:**
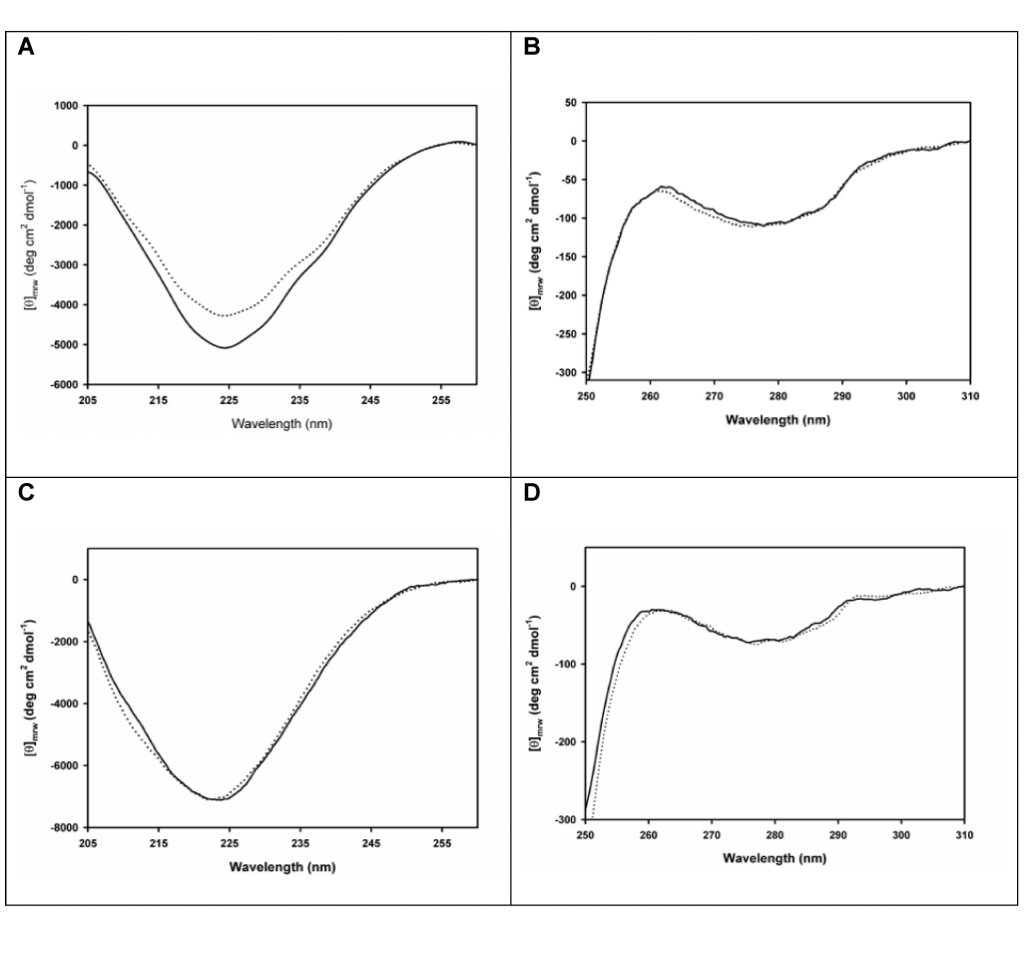
Far and near UV circular dichroism spectra of fresh and incubated lyophilized and co-lyophilized preparations of subtilisin C. CD spectra are shown for samples freshly suspended in 1,4-dioxane (solid lines) and after further incubation for 4 days (dotted lines)**(A) **Far UV CD of lyophilized subtilisin C. **(B) **Near UV CD of lyophilized subtilisin C. **(C) **Far UV CD of subtilisin C. co-lyophilized with methyl-β-cyclodextrin. **(D) **Near UV CD of subtilisin C. co-lyophilized with methyl-β-cyclodextrin.

To further probe changes in structure, including the tertiary structure, we used circular dichroism spectroscopy (CD). Spectra were recorded for both the lyophilized and the co-lyophilized powders before and after incubation in 1,4-dioxane for 4 days (Figure 3). The near UV CD of neither enzyme preparation was influenced by incubation, illustrating that the enzyme tertiary structure in both preparations remains unaffected. The far UV CD of the co-lyophilized enzyme is also unaffected, but the lyophilized powder shows a reduction in ellipticity, which was reproducible. It is unusual for a protein to show spectral changes in the far UV but not in the near UV regions, because this implies a change in secondary structure without change in tertiary structure. However, far UV CD of particulate samples is significantly affected by absorption flattening, for which a semi-empirical correction may be made [27]. The correction involves one adjustable parameter per sample, and it is possible to choose values such that the spectra for the fresh and incubated samples are the same within experimental error. However, this must remain tentative, because here we have no independent method to estimate the correction parameters. A change in absorption flattening would also be most likely to result from a change in particle structure, and none was observed in the SEM imaging studies. The spectra of the colyophilized preparation shows higher ellipticity than the lyophilized powder (Figure 3). This is most likely due to a difference in absorption flattening because the particles are different in nature. The co-lyophilized preparation was observed to disperse in dioxane better than the lyophilized powder did. It is also possible that association with chiral β-methyl cyclodextrin (the additive) induces increased CD from the protein peptide bonds.

### Enzyme pH profile

**Figure 4:**
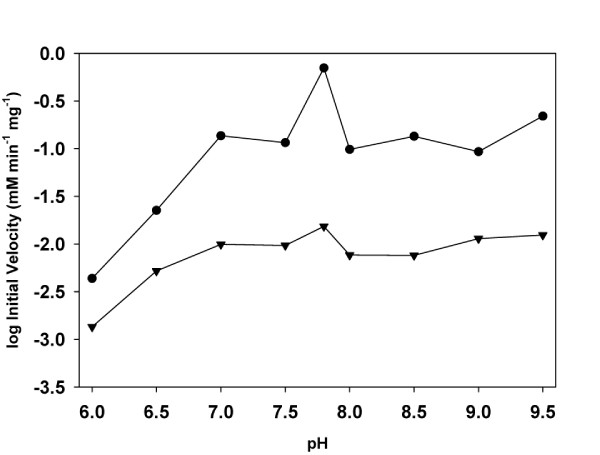
Effect of pH of co-lyophilized subtilisin C. before and after storage in 1,4-dioxane. Activity was measured for subtilisin C. co-lyophilized with methyl β cyclodextrin, then freshly suspended in 1,4-dioxane (●), and after incubation for 4 days (▼).

**Figure 5:**
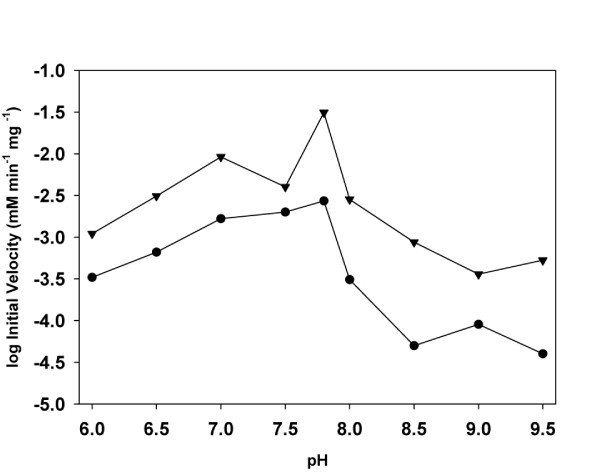
Effect of pH of lyophilized subtilisin C. before and after storage in 1,4-dioxane. Activity was measured for lyophilized subtilisin C. freshly suspended in 1,4-dioxane (▼), and after incubation for 4 days (●).

Hydrolases have been shown to exhibit optimum catalytic activity at a specific pH, where the enzyme-catalytic triad residues acquire the most favorable ionization state for catalysis. This "tuning up" of an enzyme's pH is a particularly important step in non aqueous enzymology, and it is often accomplished while the enzyme is dissolved in a buffer prior to dehydration (usually by lyophilization) before suspension in the organic solvent of choice [9]. It is also well documented that, once in an organic solvent, most enzymes "remember" their ionization state from the aqueous solution they were dissolved in prior to dehydration [8]. This is often referred to as "the enzyme's pH memory" [8]. We decided to verify if the decrease in activity observed after an incubation period could be related to the loss or to a "shift" of this pH memory optimum (subtilisin's optimum pH in aqueous buffer is 7.8). Both preparations showed a lyophilization pH optimum of 7.8, which persists after a five-day incubation period. The activity vs pH profiles (Figures 4 and 5, presented as log initial activity vs pH plots to express the possible linear correlation of the data) show that the effect of lyophilization pH is still strong after incubation (which is more evident for the co-lyophilized enzyme, Figure 4), suggesting that the activity loss is not due to a shift or loss of the enzyme's pH memory. The enantioselectivities of both preparations obtained at different pH and incubation times remained essentially constant (within a 15% error) over a wide pH range (from pH 6.5 to 8.5), but it decreased slightly at the extreme pH's.

### Active site spin labeling

**Figure 6:**
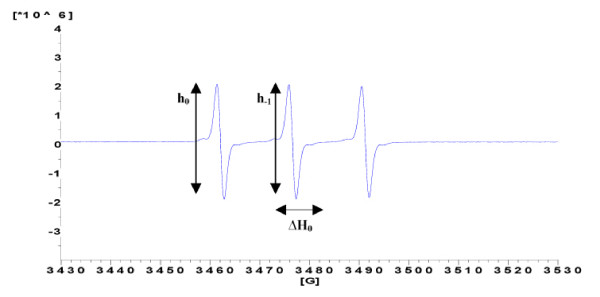
Characteristic EPR signal of the spin label in solution.

It is believed that enzyme flexibility and dynamics are directly related to its activity (and enantioselectivity) in a given solvent. Therefore, it is possible that the reduced activity observed after incubation is due to changes of the enzyme's dynamics, occurring either by solvent insertion into the enzyme, water removal or over relaxation of the active-site. Site directed spin labeling (SDSL) is emerging as a new tool for determination of structure and conformational dynamics of proteins [22]. The basic strategy of SDSL is to introduce a paramagnetic nitroxide side chain at a specific site in a protein sequence and to analyze the EPR spectrum of the spin labeled protein in terms of environmental parameters that characterize the site in protein fold. Changes of macromolecular configuration influencing the surroundings of a covalently attached spin label are reflected in the label's degree of immobilization, which can be given a quantitative expression in terms of its rotational correlation time (τ) [23]. When the label is freely tumbling in solution, its spectrum consists of three narrow lines of equal intensity. As the rate of tumbling slows from that in free solution, the three lines broaden unequally and the spectrum becomes asymmetric. From such a spectrum the rotational correlation time (τ) can be calculated. Tau is related to the time required for the label to rotate an average of 40° and is a measure of the rotational freedom of the label's "side chain" (this is defined as the entire label moiety bound to the enzyme group). A large value of τ indicates slow rotation, while rapid tumbling is associated with low values of τ. For fast isotropic motions of spin labels (τ_c _~10^-11 ^to 10^-9 ^s) the apparent rotational correlation time of spin labels can be obtained from line width and the line amplitude measured from the EPR spectra (Figure 6), using **Equation 1 **[24].

**Equation 1**: τ(s) = 6.5 × 10^-10 ^ΔH_0 _[(h_0_/h_-1_)^1/2^-1]

**Figure 7:**
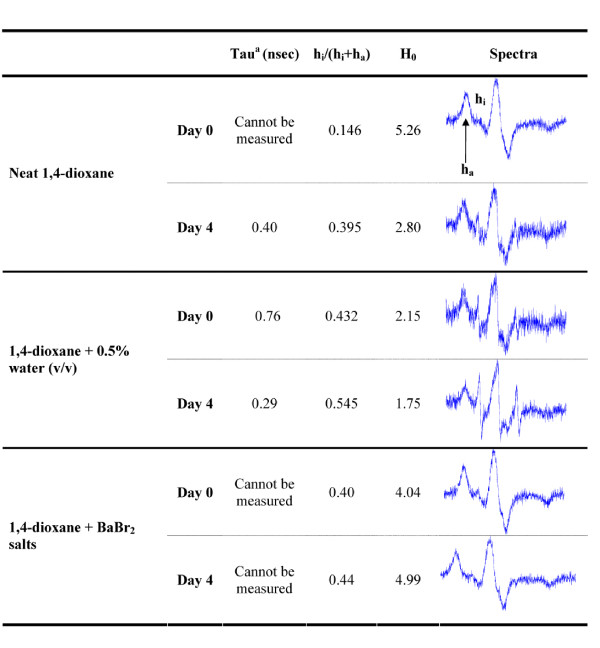
Effect of water (0.5 % v/v) and controlled water activity during incubation of 4-EFPO-TEMPO-labeled Subtilisin Carlsberg in 1,4-dioxane by EPR Spectroscopy.

**Figure 8:**
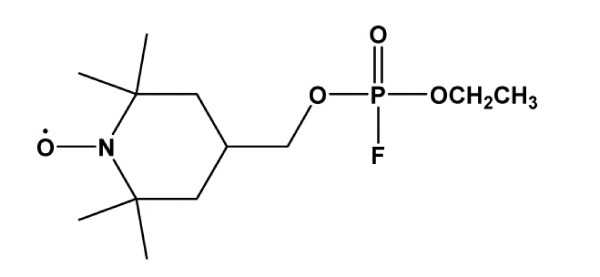
Structure of 4-Ethoxyfluorophosphinyloxy-TEMPO.

For slow molecular motions (τ > 3 × 10^-9 ^s), the line positions depend on the rate as well as the amplitude of motion. Hence, the parameter Δ**H**_**0 **_can be used as a convenient empirical measure of dynamics, including both amplitude and rate of motion (a decrease in Δ**H**_**0 **_indicates increased degree of freedom). In addition, the parameters **h**_**i **_and **h**_**a **_(spectra shown on (Figure 7) can also be used to determine the freedom of rotation of the spin label (a high value of **h**_**i**_**/(h**_**i**_**+h**_**a**_**) **indicates higher degree of freedom). For our studies, lyophilized subtilisin Carlsberg was spin-labeled at the active site with a classic inhibitor for serine proteases: 4-ethoxyfluorophosphinyloxy-TEMPO (Figure 8). After labeling, activity and active site titrations were performed to ensure the enzyme was in fact labeled at the active-site (Materials and Methods). Our results, summarized on (Figure 7), indicate that the degree of freedom (or mobility) of the spin label, bound to the enzyme's active site, increases during the five days of incubation. Although Tau could not be measured at day 0 (no incubation), Δ**H**_**0 **_and **h**_**i**_**/(h**_**i**_**+h**_**a**_**) **show that the mobility of the spin label increases after a 4-day incubation, suggesting increased flexibility of the enzyme or "over relaxation" of the active site. Besides the activity loss observed, increased mobility of the spin label seems to be the only consequence of incubation in 1,4-dioxane, and it could therefore be interpreted as the cause for the low enzyme stability. If, however, one argues that increased mobility of the spin-label relates to increased enzyme flexibility, then these results seem to be contradictory to the general belief that increasing the flexibility should increase the enzyme's activity, unless the enzyme's flexibility was already at its optimum before incubation, and increased flexibility would only cause a decrease in activity. But perhaps the most plausible explanation is that the enzyme's active-site is becoming distorted or "over relaxed" during incubation, and this results in a decrease in V_max_/K_M_, in initial activity and an increase in apparent flexibility of the spin label is observed. Since added water has been reported to increase an enzyme's flexibility [25,26], changes of hydration water from the enzyme (occurring during the incubation period) could explain the increased flexibility observed after the 5-day incubation period. To determine this, we conducted a series of incubation and EPR measurements in 1,4-dioxane + 0.5% of added water and controlling its water activity using hydrated BaBr_2 _salts.

### EPR studies of subtilisin C. in 1,4-dioxane with 0.5 % water and controlling the water activity

Spin-labeled lyophilized subtilisin C. was suspended in 1,4-dioxane, water (0.5 % v/v) was added, and the EPR spectra were recorded at day 0 (no incubation) and day 4 of storage in this solvent. The results show that in the presence of water, at day zero (no incubation), the spin label has a higher degree of mobility than that of the enzyme suspended in the neat solvent (Δ**H**_**0 **_is smaller while **h**_**i**_**/(h**_**i**_**+h**_**a**_**) **is larger – consistent with increased mobility of the spin label, (Figure 7)). Incubation of the hydrated enzyme for 5 days in this solvent shows only a moderate increase in the spin label mobility, as determined from the values of Tau, Δ**H**_**0 **_and **h**_**i**_**/(h**_**i**_**+h**_**a**_**)**. However, when these experiments were repeated controlling the water activity, no increase in enzyme flexibility was observed (Figure 7). Since hydration has a clear effect on the enzyme flexibility, and it has been well documented that added water can alter the enzyme activity, we decided to look at the effect of hydration on enzyme storage stability further.

### Enzyme storage stability in the presence of 0.5% water and controlling the water activity

**Figure 9:**
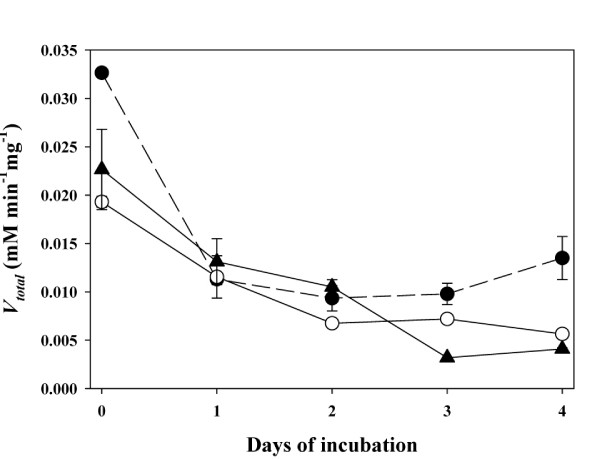
Storage stability of lyophilized subtilisin Carlsberg in 1,4-dioxane: (○) neat, (●) with 0.5% H_2_O added, and (▲) controlling water activity with BaBr_2 _salts.

**Figure 10:**
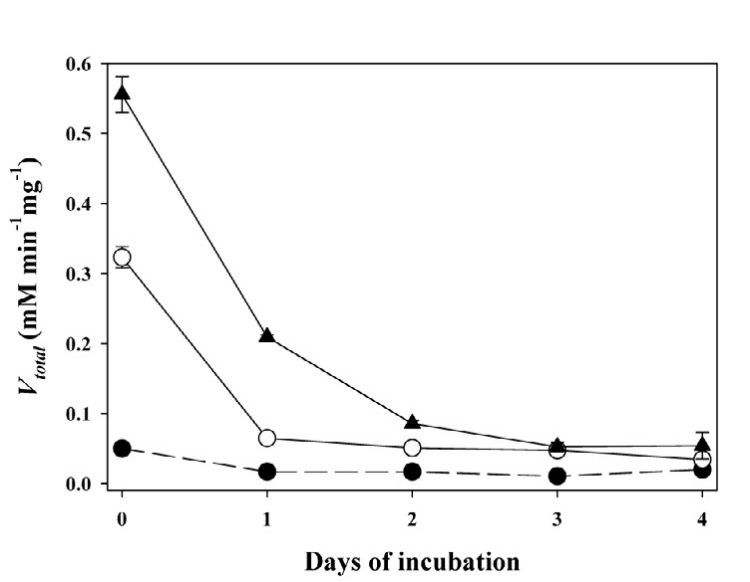
Storage stability of subtilisin Carlsberg co-lyophilized with methyl-β-cyclodextrin in 1,4-dioxane: (○) neat, (●) with 0.5% H_2_O added, and (▲) controlling water activity with BaBr_2 _salts.

To address the issue of changes of the enzyme's hydration level as a possible cause for the observed low solvent storage stability, we incubated the lyophilized and the colyophilized powders in 1,4-dioxane with 0.5% (v/v) water added to the solvent and controlling the water activity using pre-hydrated BaBr_2 _salts directly added to the solvent. The results show that regardless of the water content, both preparations lose their initial high activity during incubation. The lyophilized powder is initially activated (with the addition of water), but its activity decreases rapidly after 24 h, and a similar behaviour is observed when the water activity is controlled (Figure 9). However the activity of the colyophilized enzyme is diminished with the addition of water (as it has been previously reported) [25], and it decreases further with incubation (Figure 10). Controlling the water activity actually results in an initial increase in enzyme activity, but it also declines to approximately the same value after incubation (Figure 10). These results suggest (but do not exclude other mechanisms) that the low enzyme storage stability is not due to a hydration/dehydration effect. The fast enzyme inactivation observed when water is added should not be due to denaturation of the protein because its activity is preserved after the incubation period (the remaining activity is similar to that of the enzyme in neat solvent). This could be due to a general effect that is also observed with other enzyme formulations, such as colyophilized and pegylated, where the initial enzyme activity is high but the stability in the solvent is lower than the lyophilized powder [14,15].

## Discussion

It is clear that loss of activity is not due to any major change in structure. Other possible mechanisms include changes in the ionization state of critical residues in the enzyme, changes in the enzyme flexibility, or in the enzyme's hydration level which could also have an effect on its flexibility. Our stability data obtained with 0.5% water and with controlled water activity rules out hydration of the enzyme as the cause. Change in hydration is one factor that may affect both activity and flexibility without significant change in protein structure. Our experiments varying the water content of the organic solvent in which the enzyme is incubated are informative here. The quite different changes in flexibility seen with different water levels suggest that hydration is a major contributor to these effects.

In contrast, the much smaller effects of water level on inactivation over time suggest that this process is not so closely related to hydration (or indeed to flexibility changes). However, we certainly cannot exclude processes involving protein-bound water molecules. Activity could be affected by the loss or movement of just one or two water molecules close to the active site. Such changes might even be opposite to those in the total amount of enzyme-bound water.

The pH memory experiments tend to exclude changes in the ionization state as the cause, but the increase in K_M _observed could be the result of small but critical changes in the structure around the active-site. It is also interesting to compare these findings with the inactivation of subtilisin Carlsberg, in silica immobilised or CLEC form, during continuous operation in acetonitrile based media [27]. In this case no activity can be recovered on returning the catalyst to aqueous media [27], and CD, infrared and fluorescence measurements reveal extensive structural changes (Ganesan et al (2006), and further unpublished work). Clearly the mechanisms involved here are quite different than the storage stability studied in the present paper, perhaps the immobilized enzyme used in that study is more susceptible to structural or flexibility changes than the "native" enzyme?

## Conclusion

Our studies of secondary and tertiary structure show that the enzyme remains structurally defined during prolonged incubation in organic solvents. Changes in the enzyme hydration or aggregation do not seem to be responsible for the observed poor storage stability, although it is possible that in the presence of water, we could be observing two competing effects (activation and fast deactivation) which prevent us from clearly separating the two effects (low storage stability from a deactivation/activation effect of added water). The flexibility of the active-site spin label increases with increased water concentration, but it remains constant during the storage period when the water activity is controlled by added hydrated salts. The observed decrease in activity is also accompanied by an increase in K_M _and a decrease in V_max_, showing loss of the catalytic efficiency of the enzyme.

These results exclude structural changes, flexibility, hydration, and changes of the enzyme ionization state as possible mechanism for the observed low storage stability, but do not exclude possible depletion or rearrangement of water molecules around the active site, or small structural perturbations around the active site or movements of counter ions. Further comprehensive studies will be required to ascertain this.

## Methods

### Enzymes and reagents

Subtilisin Carlsberg (alkaline protease from *Bacillus licheniformis*, EC 3.4.21.14) and sec-phenethyl alcohol were purchased from Sigma/Aldrich (St. Louis, MO). The solvents were purchased from Aldrich in the anhydrous form (Sure/Seal bottles, water content below 0.005%). Vinyl butyrate was purchased from TCI America (Portland, OR). The spin label 4-ethoxyfluorophosphinyloxy-TEMPO (4-EFPO-TEMPO) was purchased from Toronto Research Chemicals (North York, Canada).

### Enzyme preparation and kinetic measurements

Subtilisin C. powder was prepared by 24 h lyophilization from a solution of 5 mg/mL in a 20 mM potassium phosphate buffer at pH 7.8. Colyophilization with MβCD was done by lyophilizing the enzyme from a buffer solution containing the additive at a 1:6 weight ratio of enzyme to additive. The model transesterification reaction between sec-phenethyl alcohol and vinyl butyrate was followed by gas chromatography. The GC instruments (HP 6850 and HP 6890, fitted with Chirasil CB columns, FID detectors and He as carrier gas) were calibrated with the chiral ester products of the reactions. The product peak areas and retention times were the same in the presence or absence of the substrates. The substrates (70 mM alcohol and 200 mM vinyl butyrate) and the solvent (1.0 mL) were stored over activated 3 Å molecular sieves prior to their use. All kinetic experiments were terminated before 10% of the product was formed. The enzyme enantioselectivity was determined by measuring the initial rates of enantiomeric product formation [28]. The retention times of the "R" and "S" products were obtained using samples of the pure enantiomers synthesized from the corresponding alcohol enantiomers. The enzyme enantioselectivity is equal to the ratio: [k_cat_/K_M_]_R_/[k_cat_/K_M_]_S _= V_R_[S]/V_S_[R] [29].

### Enzyme hydration and controlled activity experiments

Water activity was controlled using hydrated salt pairs as described [30]. The mixture of BaBr_2_•1H_2_O/BaBr_2 _was prepared by placing the anhydrous salt (5 g/16.8 mmol) over pure water (75.8 mg/4.2 mmol) in a sealed vessel saturated with water vapor [30]. The enzyme activity measurements performed at the resulting water activity of 0.008 were carried out as described above with the following modifications: The mixture of BaBr_2_•1H_2_O/BaBr_2 _(100 mg) was added to the solvent (1 mL) containing the enzyme and vinyl butyrate. The suspension was equilibrated by shaking at 45°C and 300 rpm for 60 min. Then, the alcohol was added and the reaction monitored as described above.

### Enzyme stability in organic solvents

Enzyme stability was determined by measuring the enzyme activity (initial velocity) as a function of the incubation time (for up to 4 days) as follows: vials containing the enzyme (lyophilized powder: 10 mg, and co-lyophilized: 1.0 mg) suspended in 1.0 mL 1,4-dioxane under N_2_, were incubated at 45°C under constant shaking (300 rpm). To measure the enzyme activity at different incubation times, the substrates were added to initiate the reaction and the kinetics followed as previously described.

### Michaelis constants (K_M_), V_max _and enzyme concentration

The apparent Michaelis constants and V_max _were determined by plotting V_s _vs V_s_/[S] measured for the model transesterification reaction between phenyl alcohol and vinyl butyrate. The alcohol concentration was increased from 1 to 100 mM, while the activated ester concentration was kept constant at 200 mM. The active enzyme concentration was determined following a published procedure [18].

### pH profile experiments

The enzyme was lyophilized and co-lyophilized from 20 mM potassium phosphate buffers of varying pH, adjusted as needed with dilute solutions of NaOH or HCl. The initial activities (for the transesterification reaction between sec-phenethyl alcohol and vinyl butyrate) at day 0 and after a 7-day incubation period were measured for each of the enzymes (prepared from buffers of different pH).

#### FTIR experiments

FTIR studies were conducted with a Nicolet Magna-IR System 560 optical bench as described [20,28,31,32]. A total of 256 scans at 2 cm^-1 ^resolution using Happ-Ganzel apodization were averaged to obtain each spectrum. Aqueous spectra were measured using spacers of <15 μm thickness. Lyophilized protein powders (lyophilized and colyophilized) were measured as KBr pellets (1 mg of protein per 200 mg of KBr). Pellets were produced using a Spectra Tech Macro-Micro KBr Die Kit and Carver 12-ton hydraulic press. Enzyme powders in organic solvents were prepared by sonication for 2 min in a sonication bath and measured in a FTIR cell equipped with CaF_2 _windows and using 100 μm thick spacer. When necessary, spectra were corrected for the solvent background and water vapor contributions in an interactive manner using the Nicolet OMNIC 3.1 software to obtain the protein's vibration spectra. Each protein sample was measured at least five times [20,32].

### Second derivatization

All spectra were analyzed by second derivatization in the amide I region (1700–1600 cm^-1^). The second derivative spectra were obtained with the derivative function of Omnic 3.1 software (Nicolet). The final protein spectrum were smoothed with an 11-point smoothing function (10.6 cm^-1^). Amide I second derivative spectra were also used to calculate the spectral correlation coefficient (see below).

#### Calculation of the correlation coefficient

Spectral correlation coefficient values (SCC) to quantify procedure-induced protein structural perturbations were calculated as described using the amide I second derivative spectra [19,28]. After correcting the spectra for the background and all water vapor contributions, the second-derivative spectra in the amide I spectral region were calculated and smoothed twice with a 10.6 cm^-1 ^function. Next, these second-derivative spectra were saved for the spectral range from 1700–1600 cm^-1 ^on identical wavenumber scales and with identical data spacing. The data for the reference spectrum (e.g. the native in aqueous medium) and the spectrum with the varied condition (e.g. after suspension in organic solvent) were imported into the program Sigma Plot and the correlation coefficient was calculated as described in detail by Griebenow et al [28]. An SCC of 1.0 indicates that two spectra being compared are the same.

### Circular Dichroism experiments

Far and near UV Circular Dichroism spectra (CD) of lyophilized and co-lyophilized subtilisin C. were studied in 1,4-dioxane, before and after incubation for 4 days as described. CD spectra were measured at room temperature in a Jasco 810 spectropolarimeter fitted with a rotating sample cell holder. Full details are described in Ganesan et al (2006). Far UV CD and near UV CD spectra were measured using cells of path length 0.01 cm and 0.5 cm, respectively. The spectra were an average of 5 scans collected at a scan speed of 50 nm/min and bandwidth 1 nm for far UV and 1.5 nm for near UV. After the measurement was completed the content of the sample cell was collected (with rinsing), air dried and dissolved in deionized water and protein concentration was estimated from UV absorption at 280 nm.

### Preparation of spin labeled subtilisin

Subtilisin Carlsberg was spin labeled at the active site Ser-221 with 4-ethoxyfluorophosphinyloxy-TEMPO (Figure 8) by the method of Morrisett and Broomfield with minor modifications in the published protocol [33]. After the reaction, the remaining free radical was removed using Sephadex G25 desalting columns (Amersham Biosciences). The spin labeled enzyme free of any unbound spin label was thereafter lyophilized and used for the EPR analysis. Active site titration using a published protocol [34] revealed a 100% reduction on the number of enzyme-active-sites (accompanied also by loss of enzyme activity), demonstrating that indeed, spin-labeling took place at the active-site. Organic solvents dried overnight over molecular sieves were used for the EPR analysis. The incubation of labeled enzyme in organic solvents was carried out at room temperature. All EPR spectra were recorded at room temperature on a Bruker EMX EPR spectrometer with a microwave power of 2.0 mW, a microwave frequency of 9.7 GHz and a sweep width of 100G (resolution 1024 points). Every spectrum was obtained from multiple scans.

## Authors' contributions

BC carried out the FTIR and incubation studies, and helped draft the manuscript. VB carried out the EPR studies. AG completed the CD studies. PH helped designing the study and drafting the manuscript. FS carried out some of the preliminary FTIR experiments and helped draft the manuscript. AF completed the pH profile and the active site titration study. KG and GB conceived the study, participated in its design and coordination, and drafted the manuscript. All authors read and approved the final manuscript.

## Figures and Tables

**Table 1:** Castillo et al., Table 1 Kinetic parameters of subtilisin C. co-lyophilized with methyl-β-cyclodextrin

	1,4-Dioxane	
	
	Day 0	Day 4
	
V_max_^a^	102 ± 3	16 ± 2
K_M_^b^	130 ± 10	319 ± 48
V_max_/K_M_	0.78	0.050

^a^mMmin^-1^, ^b^mM, both values were obtained plotting the initial velocity (V) vs (V/[S]). Model reaction: transesterification between phenyl alcohol and vinyl butyrate at 45°C.
